# Evaluating Hospital Course Summarization by an Electronic Health Record–Based Large Language Model

**DOI:** 10.1001/jamanetworkopen.2025.26339

**Published:** 2025-08-13

**Authors:** William R. Small, Jonathan Austrian, Luke O’Donnell, Jesse Burk-Rafel, Katherine A. Hochman, Adam Goodman, Jonah Zaretsky, Jacob Martin, Stephen Johnson, Vincent J. Major, Simon Jones, Christian Henke, Benjamin Verplanke, Jwan Osso, Ian Larson, Archana Saxena, Aron Mednick, Choumika Simonis, Joseph Han, Ravi Kesari, Xinyuan Wu, Lauren Heery, Tenzin Desel, Samuel Baskharoun, Noah Figman, Umar Farooq, Kunal Shah, Nusrat Jahan, Jeong Min Kim, Paul Testa, Jonah Feldman

**Affiliations:** 1 Department of Health Informatics, New York University Langone Medical Center Information Technology; 2 Department of Medicine, New York University Grossman School of Medicine; 3 Department of Population Health, New York University Grossman School of Medicine; 4 Epic Systems Corporation, Madison, Wisconsin; 5 Department of Medicine, New York University Grossman Long Island School of Medicine, Mineola; 6 Department of Emergency Medicine, New York University Grossman School of Medicine

## Abstract

**Importance:**

Hospital course (HC) summarization represents an increasingly onerous discharge summary component for physicians. Literature supports large language models (LLMs) for HC summarization, but whether physicians can effectively partner with electronic health record–embedded LLMs to draft HCs is unknown.

**Objectives:**

To compare the editing effort required by time-constrained resident physicians to improve LLM- vs physician-generated HCs toward a novel 4Cs (complete, concise, cohesive, and confabulation-free) HC.

**Design, Setting, and Participants:**

Quality improvement study using a convenience sample of 10 internal medicine resident editors, 8 hospitalist evaluators, and randomly selected general medicine admissions in December 2023 lasting 4 to 8 days at New York University Langone Health.

**Exposures:**

Residents and hospitalists reviewed randomly assigned patient medical records for 10 minutes. Residents blinded to author type who edited each HC pair (physician and LLM) for quality in 3 minutes, followed by comparative ratings by attending hospitalists.

**Main Outcomes and Measures:**

Editing effort was quantified by analyzing the edits that occurred on the HC pairs after controlling for length (percentage edited) and the degree to which the original HCs’ meaning was altered (semantic change). Hospitalists compared edited HC pairs with A/B testing on the 4Cs (5-point Likert scales converted to 10-point bidirectional scales).

**Results:**

Among 100 admissions, compared with physician HCs, residents edited a smaller percentage of LLM HCs (LLM mean [SD], 31.5% [16.6%] vs physicians, 44.8% [20.0%]; *P* < .001). Additionally, LLM HCs required less semantic change (LLM mean [SD], 2.4% [1.6%] vs physicians, 4.9% [3.5%]; *P* < .001). Attending physicians deemed LLM HCs to be more complete (mean [SD] difference LLM vs physicians on 10-point bidirectional scale, 3.00 [5.28]; *P* < .001), similarly concise (mean [SD], −1.02 [6.08]; *P* = .20), and cohesive (mean [SD], 0.70 [6.14]; *P* = .60), but with more confabulations (mean [SD], −0.98 [3.53]; *P* = .002). The composite scores were similar (mean [SD] difference LLM vs physician on 40-point bidirectional scale, 1.70 [14.24]; *P* = .46).

**Conclusions and Relevance:**

Electronic health record–embedded LLM HCs required less editing than physician-generated HCs to approach a quality standard, resulting in HCs that were comparably or more complete, concise, and cohesive, but contained more confabulations. Despite the potential influence of artificial time constraints, this study supports the feasibility of a physician-LLM partnership for writing HCs and provides a basis for monitoring LLM HCs in clinical practice.

## Key Points

**Question:**

When asked to revise hospital courses (HCs) toward a quality standard, are there differences in how many edits physicians make to large language model (LLM)–generated HCs compared with physician-generated HCs?

**Findings:**

This quality improvement study of 100 general medicine admissions found that physicians edited a statistically significantly smaller percentage of LLM-generated HCs than physician-generated HCs (mean 31.5% vs 44.8%, respectively).

**Meaning:**

These findings suggest LLM HCs were of higher quality than physician HCs and would be feasible to implement into practice.

## Teaser

This quality improvement study evaluates differences in effort among resident physicians editing large language model–generated vs physician-generated hospital courses.

## Introduction

Discharge summaries help ensure a safe transition of care for inpatients, with hospital course (HC) summarization being a key component.^1,2,3^ However, HCs have become increasingly lengthy, redundant, and inaccurate at the note reader’s expense.^4,5,6^ Rising documentation requirements,^7^ higher patient volumes and complexity,^8,9^ and multiple shift handoffs contribute to this issue. These factors lead to clinician burnout^10,11^ and maladaptive behaviors, like unedited copy-forward passages^12,13^ that degrade note quality.^14,15,16^ Although organizations have shown that educational interventions^17,18,19,20^ and electronic health record (EHR)–based tools (eg, note templates^21^) can improve documentation quality and author or recipient satisfaction, significant opportunities remain.

Large language models (LLMs) are adept at summarizing clinical information^22,23,24,25,26^ and may improve documentation quality and physicians’ documentation time requirements.^27,28,29,30,31^ LLMs can synthesize large amounts of clinical data,^32,33,34,35^ including documentation, into HCs that rival the quality of physicians.^5,6,36,37,38^ However, prior evaluations of LLM-generated drafts do not address the clinical practice complexities of clinician-LLM interactions, akin to deeming a tool efficacious without understanding its effectiveness in clinical practice.^39,40,41^ To date, no study has evaluated the feasibility of partnering physicians with an EHR-embedded LLM to create high-quality HCs.

In December 2023, Epic Systems Corporation customers were offered the opportunity to test an EHR-embedded LLM-enabled HC summarization tool. To assess the tool’s feasibility, we developed a 4Cs (complete, concise, cohesive, and confabulation-free) HC quality standard (Table 1; eMethods in Supplement 1). This standard measures HC quality and serves to guide HC composition and editing. The primary objective of our study was to compare the editing effort required by time-constrained resident physicians to improve LLM- vs patients’ actual clinician-generated HCs. Secondarily, attending physicians compared documentation quality between edited LLM and physician HCs using the 4Cs HC quality standard. We hypothesized that improving LLM HCs would require similar editing effort as physician HCs, demonstrating the feasibility of implementing a physician-LLM partnership for HC composition.

**Table 1.** Hospital Course Quality Standard Components

Quality component	Description
Complete	The hospital course should provide a thorough account of the patient’s initial presentation, inpatient treatment, and resolution at discharge
Concise	The hospital course should include only relevant information, avoiding unnecessary details
Cohesive	The hospital course should present an easy-to-follow narrative that clearly delineates the clinical thought process and the patient’s journey
Confabulation-free	The hospital course should be accurate and free from hallucinations or erroneous entries

## Methods

This quality improvement study evaluates the editing effort required by time-constrained residents to improve LLM- vs patients’ actual physician-generated HCs. Attending physicians secondarily rated pairs of edited HCs using the 4Cs HC quality standard (eFigure 1 in Supplement 1). The study was conducted as an operational prepilot program to implement an EHR-embedded LLM HC tool and was exempt from institutional review board review based on the New York University Langone Health (NYULH) self-certification protocol. All study procedures complied with institutional ethical standards and those set by the Declaration of Helsinki and are reported using the Standards for Quality Improvement Reporting Excellence (SQUIRE) reporting guideline^42^ and Transparent Reporting of a Multivariable Prediction Model for Individual Prognosis or Diagnosis (TRIPOD)-LLM reporting guideline^43^ checklists for reporting quality improvement studies and those involving LLMs, respectively.

### Study Setting

The study took place at NYULH, a large academic health care system with 3 main hospital campuses. NYULH HC notes are configured to allow clinicians to continuously document a running summary of the patient’s admission, which autopopulates into our discharge summary template. The expectation is that these notes are edited for content and clarity by discharge.

### Developing the 4Cs Hospital Course Quality Standard

A panel of 8 physicians (J.F., K.H., J.Z., J.A., A.G., J.R., W.S., and J.K.) with expertise in note quality and quality improvement methodology developed the 4Cs HC quality standard (Table 1; eMethods in Supplement 1) to assess the structural components of HC quality. The process was guided by the Messick validity framework^44^ and involved 4 steps. In the first step, content validation, the panel distinguished the HC from the broader discharge summary and evaluated existing NYULH HCs. After comprehensive literature review, they identified constructs of high-quality HCs and their unique elements. In the second step, response process, faculty outside the panel provided feedback on the 4Cs HC quality standard. Participants used a think-aloud protocol to verbalize their thought processes while applying the standard. Over multiple iterations, residents edited HCs using this standard and reviewed their work with supervising attending physicians before they both provided feedback. This process ensured the standard was practical, detailed, and aligned with the intended construct. In the third step, internal structure, 3 attending physicians evaluated a random sample of 24 HCs on whether they met the requirements for each structural component. The intraclass correlation coefficient (ICC) values indicated good reliability for completeness (ICC = 0.83), conciseness (ICC = 0.83), and cohesiveness (ICC = 0.76). Due to their anticipated rarity, ICC for confabulations was not assessed. In the fourth step, consequences, the tool was evaluated for both intended and unintended consequences. Editors (residents) and reviewers (attending physicians) provided targeted feedback on the tool’s ability to guide HC evaluation and whether each component was interpretable and discrete. Furthermore, HCs improved when edited toward the 4Cs HC quality standard.

### EHR-Embedded Hospital Course Tool

We used an EHR-embedded LLM HC tool that functioned with a prompt ensemble using both GPT-3.5 (turbo version 1106; hereafter referred to as model 1), GPT-4 (version 0613; model 2), and sometimes GPT-4-32k (version 0613; model 3), depending on whether the token limit of model 2 was exceeded. The user interface contains various selectable parameters (eg, preferred length).

The LLM HC tool first summarized all eligible notes (eTable 1 in Supplement 1) over 500 characters in each patient’s EHR with model 1. Note summaries were combined into daily summaries and then daily deltas (summaries of differences between consecutive hospital days) using model 2. The LLM HC tool inputs thus included the presummarized admission note, the first day’s summary, each daily delta, and patient information dictated by default parameter settings chosen after iterative testing by physicians (eTable 2 in Supplement 1).

### Engineering of the Downstream User Prompt by the Study Team

We developed a prompt that aligned with the 4Cs HC quality standard to generate an LLM HC draft from the previously mentioned inputs. The prompt development team included a PhD data scientist (V.M.), 3 board-certified physician informaticists (J.F., J.A., and L.O.), a clinical informatics fellow board-certified in internal medicine (W.S.), and 2 board-certified hospitalist attendings (K.H. and J.Z.). Over 6 weeks, the team iteratively tested and refined their approach (ie, prompt engineering) while individually keeping experiment journals and documenting test results for each iteration. Physicians tested the tool on patients they recently treated without submitting outputs into their official medical record. Group feedback sessions revealed opportunities to improve outputs through parameter selections and prompt engineering. After 6 weeks, the best-performing HC generation prompt (which included few-shot learning, a technique where LLM outputs are guided by examples) was chosen (eMethods in Supplement 1) along with default parameters (eTable 2 in Supplement 1).

### Data Collection and Participants

We randomly selected 100 general internal medicine hospitalizations with a length of stay (LOS) of 4 to 8 days during December 2023 that were evenly distributed across our 3 main campuses. The LOS range was selected to focus on the typical hospitalization and to avoid the models’ context window limitations. 100 admissions were selected based on the resources available to complete HC editing and rating, resulting in a post hoc power of more than 90% to detect an effect size of 0.51 at alpha = .05.

We recruited 10 internal medicine resident volunteers from the NYU Grossman School of Medicine to edit HCs after they provided verbal consent and received training on the 4Cs HC quality standard. Residents could opt out at any time.

To simulate clinical time constraints, each resident reviewed each patient’s EHR (excluding the discharge narrative) for 10 minutes before editing the physician or LLM HC in random A/B order blinded to author type. Furthermore, they were given only 3 minutes to improve each HC toward the 4Cs HC quality standard.

### Survey

To evaluate edited HCs, we recruited 8 hospitalist attendings without prior exposure to the study or the 4Cs HC quality standard. After 10 minutes of EHR review (excluding the discharge narrative), each hospitalist evaluated 25 pairs of resident-edited HCs, blinded to author type. Each HC pair was comparatively evaluated by 2 random hospitalists on each component of the 4Cs HC quality standard using a 5-point Likert scale (ie, for each item: HC A is significantly better; HC is A slightly better; A and B are even; HC B slightly better; HC B significantly better) within REDCap (eFigure 1 in Supplement 1).^45^

### Editing Metrics

We quantified the amount of editing performed on the original HCs (total edits) using Levenshtein distance (Levenshtein package version 0.23.0), an algorithm that counts the minimum number of character (ie, letter, number, or symbol) changes required to convert one text to another (eTable 3, eTable 4 in Supplement 1).^46,47^ To fairly compare the HC pairs, we needed to control total edits for the statistically significantly different lengths of LLM vs physician HCs. We define percentage edited (ie, normalized Levenshtein distance) as our primary outcome, which was calculated by dividing total edits by the length in characters of whichever document contained more characters, the original or edited HC.^48,49,50^

To further measure editing effort, we quantified the extent to which the original HC’s meaning was changed. We first used an LLM fine-tuned on nearly 2 million clinical notes (BioClinicalBERT, MIMIC-III database^51^) to assign values to each HC that represent their meaning in a high-dimensional space (ie, document embeddings^52^). The difference in meaning between any 2 documents can be calculated with a metric called cosine similarity,^52,53^ which ranges from 0-1 where 1 is maximal similarity and has been reported as a percentage in peer reviewed literature.^54^ For example, cosine similarity is higher when comparing clinical notes about pneumonia and influenza than notes about pneumonia and knee replacement. To assess the percentage of the original HC’s meaning that was altered by the editing process, we define semantic change as the inverse of cosine similarity between the pre-edited and postedited HCs. See eTables 3 and 4 in Supplement 1 for a plain language introduction to these metrics.

### Statistical Analysis

Statistical analysis was conducted using Python version 3.9.16 (Python Software Foundation) from September to December 2024. We employed a priori significance levels of *P* < .05 for 2-sided tests of the null hypothesis that physician HCs and LLM HCs would require equal editing effort to meet the 4Cs HC quality standard and would be perceived as equivalently complete, consistent, coherent, and confabulation-free. We used Wilcoxon signed-rank tests, robust to outliers and nonnormal distributions,^55^ to evaluate for differences between dependent HC pairs. *P* values for the 11 secondary analyses were split into families of 6 and 5 for editing and survey outcomes, respectively, before undergoing a Šidák correction.^56^ No survey responses were excluded due to incomplete or missing data. Survey scores were standardized so that 0 represented an equal score for each A/B comparison. If the edited physician HC was perceived as slightly or significantly better, the score was set to −5 or −10, respectively. Conversely, scores were set to 5 (slightly better) or 10 (significantly better) for the edited LLM HC. Each of the 4Cs components were analyzed separately, and then as a composite score calculated from their sum.

When developing the 4Cs HC quality standard, all 3 reviewers answered yes or no on whether a sample of HCs were concise, complete, or cohesive. We thus calculated intraclass correlation (ICC) using a 2-way mixed effects model for average fixed raters.^57^ For the 5-point Likert survey questions, we calculated percentage agreement, agreement within 1 scale point, and visualized the frequency of score combinations between the 2 reviewers in a contingency table (eTable 5, eFigure 2 in Supplement 1). Given reviewers’ agreement, we treated all 200 survey results as independent evaluations.

## Results

The 100 general internal medicine hospitalizations had a mean (SD) length of stay of 5.7 (1.3) days evenly distributed across our 3 main hospitals. LLM-generated HCs contained more words than physician-generated HCs in both their original (mean [SD] LLM, 281.9 [31.0] vs physician, 225.9 [134.9] words; *P* < .001) and postedited formats (mean [SD] LLM, 237.2 [56.6] vs physician, 207.2 [95.1] words; [data]; *P* < .001).

### Analysis of the Editing Process

**Figure 1.**
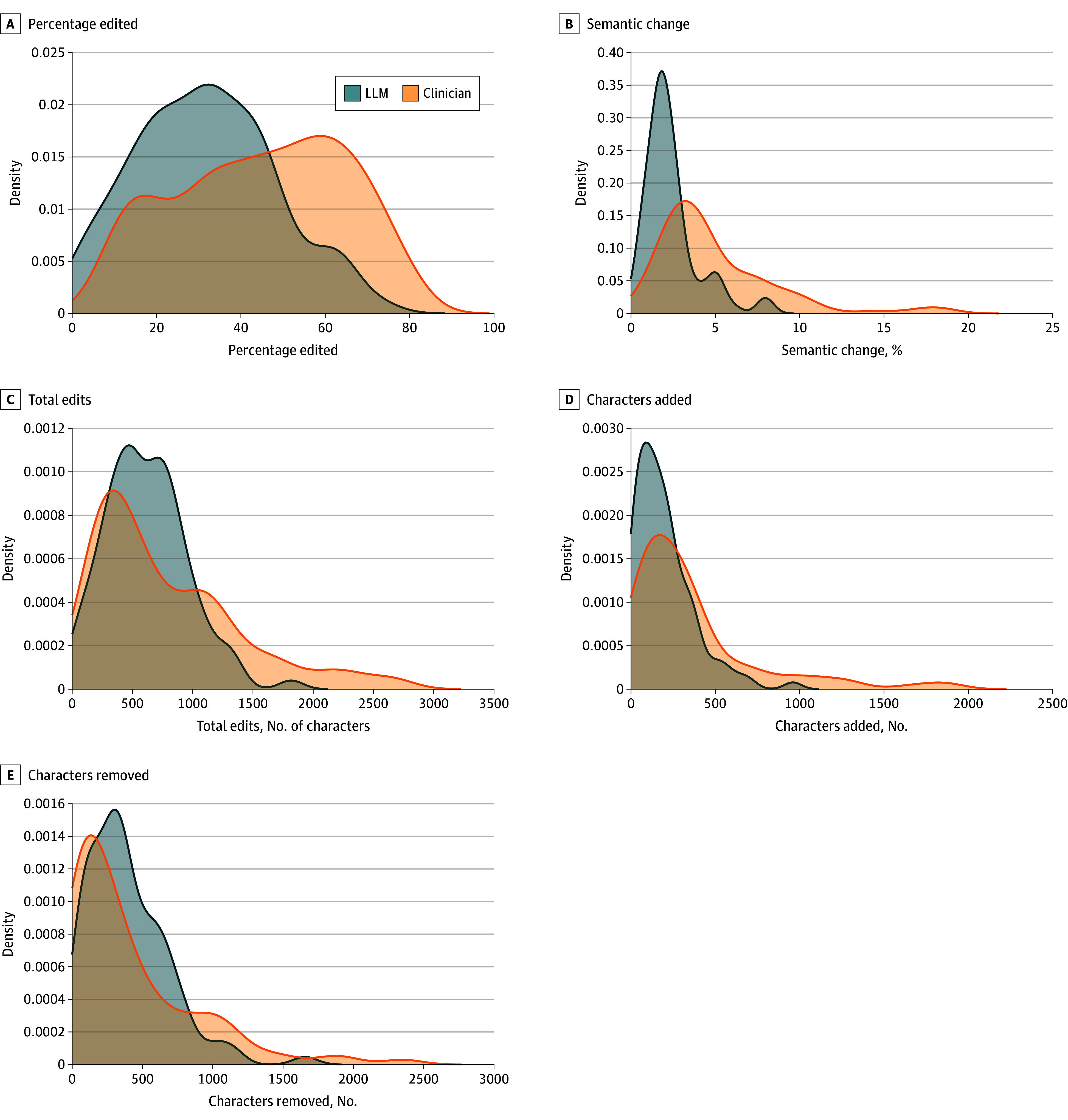
Density Plots Comparing the Distributions of Editing Metrics for Large Language Model (LLM) and Clinician Hospital Courses

LLM HCs required less editing than physician HCs (mean [SD] percentage edited LLM, 31.5% [16.6%] vs physician, 44.8% [20.0%]; *P* < .001) and exhibited less variation in the proportion edited (Table 2; Figure 1A). Therefore, on average, about one-third of each LLM HC was edited compared with half of each physician HC.

**Table 2.** Comparison of Editing Metrics Between the Pre-Edited and Postedited Hospital Courses (HCs)

Edit measure compared with unedited HC	Mean (SD)		*P* value
	LLM-Generated HC	Physician-Generated HC	
Amount edited (normalized Levenshtein distance), %	31.5 (16.6)	44.8 (20.0)	<.001
Semantic change (higher more change), %	2.4 (1.6)	4.9 (3.5)	<.001
Total character edits (Levenshtein distance)	616.6 (336.7)	782.2 (611.0)	.63
Total characters added	196.9 (172.2)	358.9 (383.8)	<.001
Total characters removed	395.7 (287.9)	422.1 (463.7)	.99

Abbreviation: LLM, large language model.

A smaller semantic change occurred when residents edited LLM HCs vs physician HCs (mean [SD] LLM, 2.4% [1.6%] vs physician, 4.9% [3.5%]; *P* < .001). Edited LLM HCs were thus more similar in meaning to unedited LLM HCs than edited physician HCs were to their unedited counterparts (Figure 1B).

Total character edits were 21% lower for LLM HCs, but not statistically significantly different (mean [SD] LLM, 616.6 [336.7] vs physician, 782.2 [611.0] characters; *P* = .63) (Figure 1C). Residents added 82.3% more characters to physician HCs, a statistically significant difference (mean [SD] LLM, 196.9 [172.2] vs physician= 358.9 [383.8] characters; *P* < .001) attributable to the distribution’s rightward skew (Figure 1D). There was no difference in total characters removed (mean [SD] LLM, 395.7 [287.9] vs physician, 422.1 [463.7] characters; *P* = .99).

### Attending Ratings of HC Quality

**Figure 2.**
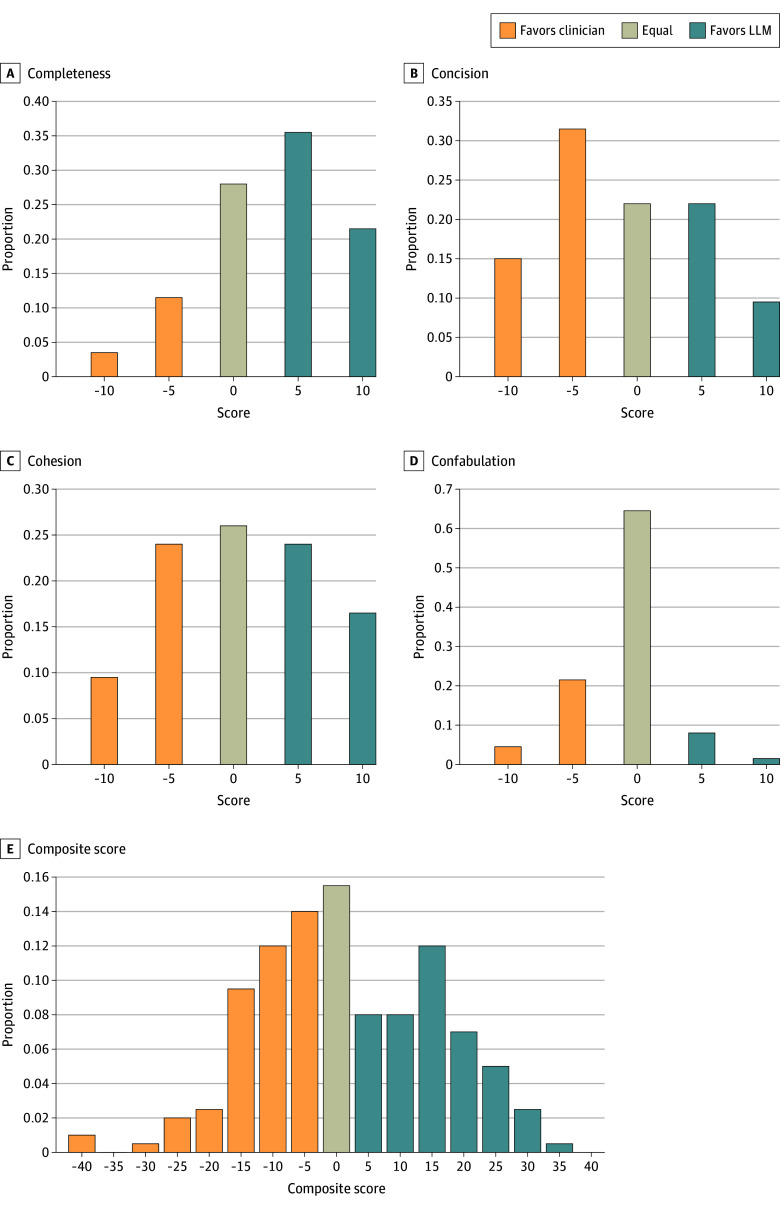
Components of the 4Cs Hospital Course Quality Standard and Composite Scores A score of −10 indicates physician HC rated significantly better, −5 indicates physician HC rated slightly better, 0 indicates HC pair equal, 5 indicates LLM HC rated slightly better, 10 indicates LLM HC rated significantly better.

Attending physicians gave high ratings for completeness, conciseness, and cohesiveness of LLM-authored HCs (Table 3). For the complete component, edited LLM HCs scored statistically significantly higher (mean [SD] difference LLM – physician on a 10-point bidirectional scale, 3.00 [5.28]; [data]; *P* < .001) and were over 6 times more frequently rated as significantly better than edited physician HCs (Figure 2A). Edited physician HCs scored statistically better on being confabulation-free (mean [SD] −0.98 [3.53]; *P* < .001), though there was no difference on 129 out of 200 evaluations (64.5%). Among the 52 out of 200 evaluations (26.0%) where edited physician HCs were considered more confabulation-free than edited LLM HCs, 43 out of these 52 evaluations (82.7%) indicated that the physician HCs were slightly more confabulation-free. The remaining 9.5% of evaluations found that edited LLM HCs were freer of confabulations than physicians. The composite (mean [SD] difference between LLM and physician on 40-point bidirectional scale, 1.70 [14.24]; *P* = .46) (Figure 2B), concise, and cohesive scores did not differ. Interrater reliability for the survey questions was good for within 1 Likert level (63%-88% agreement) but only fair for exact rating agreement (26%-47%) for each of the components (eTable 3, eFigure 2 in Supplement 1).

**Table 3.** Attending Physician Ratings of Edited HCs by Each Component of the 4Cs Hospital Course Quality Standard (Higher Favors Edited LLM-Authored HCs)

	Mean difference (SD)	*P* value
Complete^a^	3.00 (5.28)	<.001
Concise^a^	−1.02 (6.08)	.20
Cohesive^a^	0.70 (6.14)	.60
Confabulation-Free^a^	−0.98 (3.53)	.002
Composite^b^	1.70 (14.24)	.46

^a^ Range: reported as mean (SD) difference of LLM minus physician on a scale from −10 (physician preferred) to 10 (LLM preferred).

^b^ Range: reported as mean (SD) difference of LLM minus physician on a scale from –40 (physician preferred) to 40 (LLM preferred).

## Discussion

Physician- and LLM-generated HCs required substantial editing to approximate the 4Cs HC quality standard. LLM HCs required significantly less editing and preserved more semantic meaning than physician HCs. Although LLM HCs were 25% longer than physician HCs, residents performed over 20% less total edits on them. These findings suggest LLM HCs more closely represent our quality standard than patients’ actual physician HCs.

Attending physicians perceived the edited HC pairs to be equal in overall composite quality. Although edited LLM HCs were 30 words longer than edited physician HCs on average, they were considered similarly concise. Edited LLM HCs were also deemed more complete because they more consistently included summaries of patients’ presentation, treatment, and resolution. EHR-embedded LLMs thus appear to encourage time-constrained physicians to include the critical components of a high-quality HC while maintaining readability.

However, edited LLM HCs were rated to have more confabulations by about 1 point on a 10-point bidirectional scale, though physician HCs were usually rated as only slightly better, indicating most confabulations were minor. In group discussions, attending physicians attributed many LLM confabulations to awkward language and errors with event sequencing. Despite speculation,^39,40,41^ this is the first evidence that LLM drafts may influence time-constrained clinicians to make more documentation errors than the current state.

This work adds nuance to existing literature on LLMs’ comparable performance to physicians on HC summarization.^5,6,36,37,38^ Our main contribution was quantifying the physician-LLM partnership with editing metrics. Health systems can emulate these methods and benchmarks to study how physicians interact with LLM-generated content. Further, by studying EHR-embedded LLMs, we highlight the challenges of clinical data (eg, deciding what data types to include) that future implementors of this technology must consider. Finally, the 4Cs HC quality standard could aid health systems or physicians aiming to assess HC quality.

Since completing this quality improvement study, we have partnered with Epic to implement updates that enhance the efficacy and safety of LLM HCs. Facts within LLM HC drafts now contain citations to clinical notes so physicians can efficiently factcheck them. Context window limitations of model 2 and model 3 necessitated a workaround, where LLM inputs included summaries of summaries, which frequently led to chronological inaccuracies. Newer models have longer context windows, eliminating the need for this workaround.

Future studies should examine in real clinical settings whether EHR-embedded LLM HC summarization tools improve documentation quality and physician burden. Our work characterizing edit distance against a quality standard can serve as a benchmark for expected physician-LLM interaction. We have begun establishing the clinical relevance of editing metrics (eg, semantic change) by pairing them with physician evaluations, but further validation is needed to determine clinically significant editing thresholds. Future research should characterize the type and severity of documentation errors allowed by physicians editing LLM-generated content. Evidence is also needed that examines whether interventions like attestation workflows and disclaimers about LLM hallucinations improve documentation quality. Clinicians partnering with LLMs will require extensive education on best practices, which aren’t yet established but should detail key areas to review (eg, 4Cs HC quality standard), strategies for minimizing errors, and expected editing practices. Finally, document quality could be monitored at scale with LLMs as judges guided by validated evaluation frameworks.

### Limitations

Our study has several limitations. Generalizability is limited by the small sample size of 100 admissions with hospital stays between 4 to 8 days in a specific setting where residents and advanced practice physicians write HCs with attending supervision. The impact of blinding the editors and evaluators is limited by their potential recognition of LLM generated outputs. By limiting editing and EHR review time to 3 and 10 minutes, respectively, we may have been overly constrictive and inflated the allowed errors. Our study relied on specific versions of model 2, and potential biases were not fully explored, including differences between outputs of model 2 and model 3. During the study, LLM input length limitations resulted in workarounds that impacted output quality and are not relevant today. Additionally, we did not assess the clinical significance of differences in the 4Cs between edited HC types, specifically confabulations and their potential for harm.

## Conclusions

In this quality improvement study, we found physicians can feasibly partner with an EHR-embedded LLM HC summarization tool to potentially alleviate their documentation efforts and improve HC quality. Compared with physician HCs, LLM HCs required less editing to approximate our 4Cs HC quality standard and were deemed more or similarly complete, concise, and cohesive by attending physicians. Edited LLM HCs were perceived to have more confabulations, although they were not typically severe and may have resulted from the LLM’s training corpus, the clinical documentation processing algorithm, the user prompt, automation bias, or study design. While these tools have exciting potential, their integration into clinical practice requires EHR vendors and health systems to support physicians with education, local validation, and safety guardrails.

## Notes

Supplement 1. **eFigure. **Flowchart Describing Methods for Data Processing and Evaluation **eTable 1. **Note Types Included for Summarization, All Remaining Note Types Not Listed Were Considered “Other” Notes **eTable 2.** Default Parameter Selections Chosen for EHR-Embedded Hospital Course Tool **eTable 3.** Plain Language and Technical Definitions of Editing Metrics **eTable 4.** Guide to Interpreting Editing Statistics: Scenarios and Corresponding Statistical Patterns **eTable 5.** Percentage Rater Agreement on the 4Cs HC Quality Standard Survey Questions **eFigure 2.** Reviewer Score Combinations on Each of the 4Cs HC Quality Standard Survey Questions

Supplement 2.
Data Sharing Statement
