# Supplementary material for: Evaluating Hospital Course Summarization by an Electronic Health Record–Based Large Language Model
Source: JAMA Netw Open. 2025 Aug 13;8(8):e2526339. doi: 10.1001/jamanetworkopen.2025.26339 (PMC12351420; doi:10.1001/jamanetworkopen.2025.26339)
Supplement: Supplement 1. — eFigure. Flowchart Describing Methods for Data Processing and Evaluation eTable 1. Note Types Included for Summarization, All Remaining Note Types Not Listed Were Considered “Other” Notes eTable 2. Default Parameter Selections Chosen for EHR-Embedded Hospital Course Tool eTable 3. Plain Language and Technical Definitions of Editing Metrics eTable 4. Guide to Interpreting Editing Statistics: Scenarios and Corresponding Statistical Patterns eTable 5. Percentage Rater Agreement on the 4Cs HC Quality Standard Survey Questions eFigure 2. Reviewer Score Combinations on Each of the 4Cs HC Quality Standard Survey Questions [file jamanetwopen-e2526339-s001.pdf]

Supplemental Online Content

Small WR, Austrian J, O'Donnell L, et al. Hospital course summarization by an electronic health record–based large language model. *JAMA Netw Open*. 2025;8(8):e2526339. doi:10.1001/jamanetworkopen.2025.26339

eFigure 1. Flowchart Describing Methods for Data Processing and Evaluation

eTable 1. Note Types Included for Summarization, All Remaining Note Types Not Listed Were Considered "Other" Notes

eTable 2. Default Parameter Selections Chosen for EHR-Embedded Hospital Course Tool

eTable 3. Plain Language and Technical Definitions of Editing Metrics

eTable 4. Guide to Interpreting Editing Statistics: Scenarios and Corresponding Statistical Patterns

eTable 5. Percentage Rater Agreement on the 4Cs HC Quality Standard Survey Questions

eFigure 2. Reviewer Score Combinations on Each of the 4Cs HC Quality Standard Survey Questions

This supplemental material has been provided by the authors to give readers additional information about their work.

© 2025 Small WR et al. *JAMA Network Open*.

eFigure 1. Flowchart Describing Methods for Data Processing and Evaluation

Legend: The left side describes the high-level steps for data processing and evaluation, along with further description of the primary outcome and a screenshot of the survey on the right.

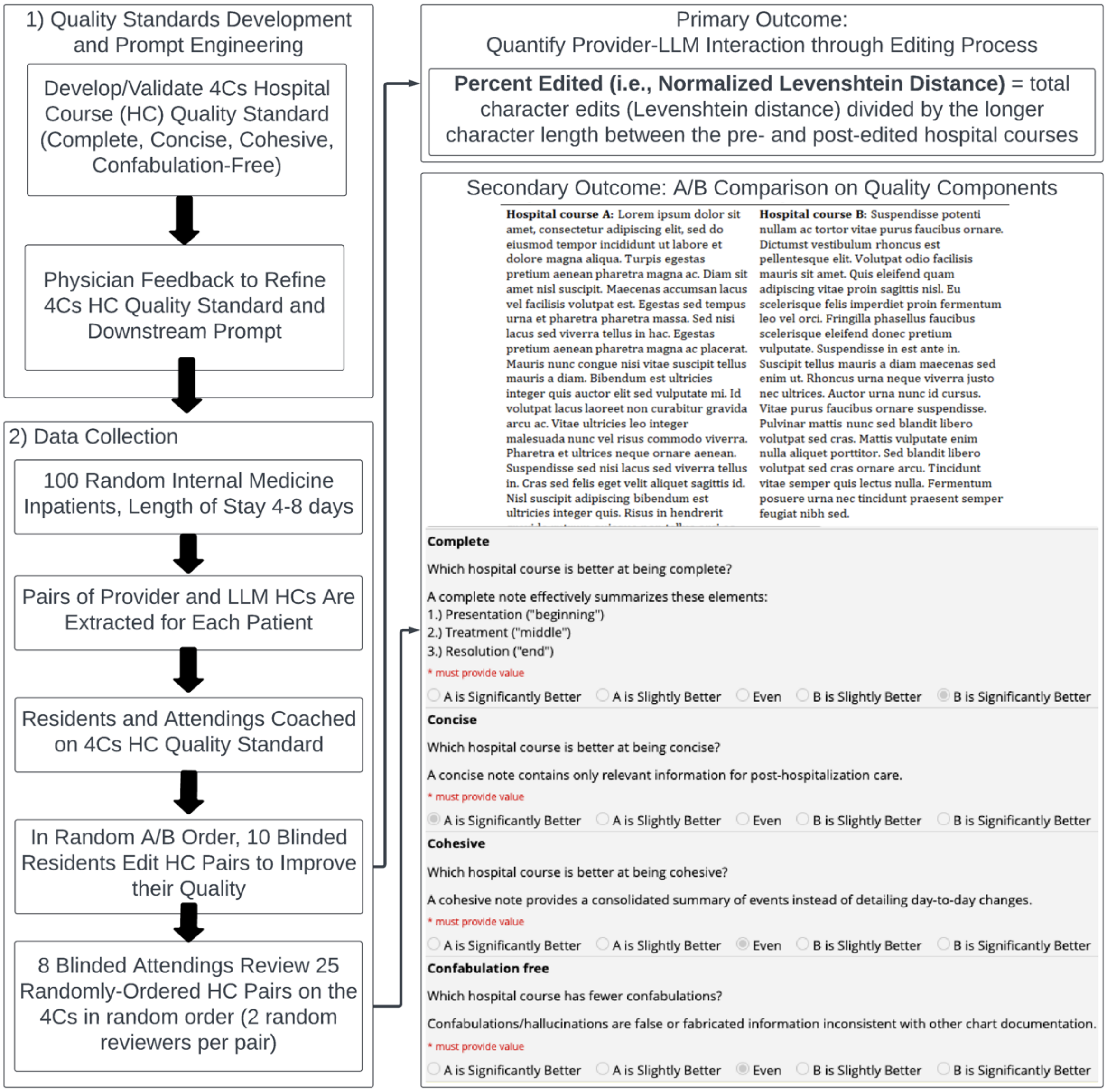

© 2025 Small WR et al. *JAMA Network Open*.

eMethods 1: Documentation quality governance at NYULH and 4Cs Hospital Course Quality Standard

I. Documentation Committee Membership
II. Committee Guiding Principles
III. Appointment of HC Standard Development Panel
IV. Literature Review and Thought Process
V. 4Cs Quality Components Description
VI. 4Cs Hospital Course Quality Standard
VII. Few Shot Learning Examples Included in the LLM Prompt

In 2020, NYU Langone established an Enterprise-Wide Documentation Standards Committee to set the objectives and strategies to enhance clinical documentation. The committee consisted of clinical, operational, quality, and informatics leaders from each hospital campus in the health system. It has extensive experience using AI and standardization to improve the quality of clinical documentation.

I. Documentation Committee Membership

- Office of the CMO for each campus
- Health System Surgeon in Chief
- Vice Chairs: Departments of Medicine, Surgery, Emergency Medicine, Pediatrics, Neurology, Physiatry, Orthopedics
- CMIO and Clinical Informaticists
- MCIT Clinical Systems
- Quality and CDI Medical Directors
- Compliance and Regulatory Affairs
- Clinical Champion Appointed from Each Hospital Service
- Frontline Staff (Residents, Chief Residents, PA/NPs)

II. Committee Guiding Principles

- Clear and complete notes to support world-class care
- Accurate and concise – everything that's needed and nothing more
- Evidence of the highest quality and accurate complexity of the care provided
- Supports the joy of practice for both the reader and the author
- Thoughtful decision-making and clinical assessment

© 2025 Small WR et al. *JAMA Network Open*.

- Maintains high reliability including contingency and escalation plans
- Catalyst for continuous improvement
- Is the product of collaborative clinical care
- Transparent and patient-centered
- Consistent across the continuum of care
- Achieves legal, external reporting, revenue, and regulatory obligations

III. Appointment of HC Standard Development Panel

To develop a hospital course standard, a panel of 8 attending physicians with expertise in note quality, QI methodology, assessment, and feedback was assembled.

- The panel included:
  - Vice Chair of Medicine and Director of Hospitalist Medicine Division
  - Health System Director of Transformation and Informatics and Residency Program Associate Director
  - Gastroenterology Fellowship Director and Campus Medical Director of Quality
  - Associate Service Chief of Medicine
  - Clinical Informatics Fellow and Hospitalist
  - An Attending Hospitalist, ED, and Primary Care Physician, all of whom depend on the quality of hospital course notes as part of their routine clinical practice.
- The panel's final recommendations were reviewed and approved by the documentation committee.

IV. Literature Review and Thought Process

The process began with a comprehensive literature review of existing tools, revealing no well-established framework for hospital course quality but providing insights from various note quality standards and existing literature on discharge summarization.

- The committee examined published hospital courses within the organization, noting their strengths and weaknesses.
- Evaluated Joint Commission Composite Elements for discharge summarization, noting which were applicable to hospital course generation in our current workflow.
- Developed themes and defined the scope for inclusion and exclusion within the standard.

V. 4Cs Quality Components Detailed Description

COMPLETE

Does this note contain the following elements:

1. Appropriate Presentation Summary - "Beginning"

© 2025 Small WR et al. *JAMA Network Open*.

- A chief complaint
- Supporting Data
- Diagnosis or Differential Diagnosis

Example:

61 yo M with DM, HTN presents with SOB and fever x 3 days. On presentation he was febrile 101.4, O2 sat= 90% on RA. WBC ct = 15.9K. CT chest showed LUL PNA. RVP was negative. He was diagnosed with sepsis from LUL PNA.

2. Appropriate Treatment Summary - "Middle"

- Initial management for primary diagnosis: state the medical therapies or procedures the patient was initially treated with. Include whether they were effective or not.
- Subsequent management: what changes were made to the initial therapy, if any? Mention improvements (or lack thereof) in the patient's clinical status (give examples of subjective and objective findings such as improvements in abnormal labs or vital signs).
- Hospital Acquired Conditions or Complications if applicable.

Example:

Patient was treated with ceftriaxone and azithromycin and placed on 2L NC. His culture data remained negative, including atypical organisms. Overnight, patient became delirious and sustained a fall without injury while going to the bathroom. On hospital day 2, he defervesced, his WBC dropped to 10.2K and no longer required supplemental oxygen. EKG showed QTc prolongation and azithromycin was changed to IV doxycycline.

3. Appropriate Resolution Summary – "End"

- Transition Description: Details of the patient's transition from an illness state requiring acute inpatient hospital care to post-acute care (include whether medications used to treat the primary diagnosis were discontinued, changed to an oral format, or continued after).
- Post-acute Care Treatment and Follow-Up Plan: The plan for continued treatment after discharge.

Example:

On HD3, the patient was converted to PO doxycycline monotherapy because they remained afebrile for 2 days, no longer required supplemental oxygen, and physical therapy deemed them to have a normal functional status. At discharge the patient will require 3 additional days of outpatient therapy.

© 2025 Small WR et al. *JAMA Network Open*.

CONCISE

Does the note contain only relevant information for post-hospitalization care?

Avoids including unnecessary details that would not be relevant post-hospitalization.

Examples:

- Unnecessary PMHx or HPI
- Non-contributing lab and imaging results.
- Temporary chronic medication adjustments, e.g., transient insulin increase not continued at discharge.
- Routine IV fluids during hospital stay.
- Temporary diet changes not continued post-discharge.
- Routine prophylaxis decisions, e.g. Lovenox for DVT prophylaxis.
- Routine hospital screening tests e.g MRSA nasal swab.
- Routine electrolyte repletion.

COHESIVE

Does the note provide a cohesive summary, rather than detailing day-to-day changes?

Does it sound like a single provider presenting the case to a primary care doctor?

Examples:

Avoid: On admission, patient received 1 unit PRBCs and Hgb increased to 8.3. On hospital day 2, patient's hemoglobin was 7.5. On day 3 of hospitalization, patient was transfused again with 1 unit PRBC. On day of discharge, patient's hemoglobin was 9.3.

Include: The patient required two units of PRBC due to a GI bleed. At the time of discharge, the patient's hemoglobin was stable at 9.3.

Avoid: HPI then ED course then daily update.

Include: A combined summary for the whole hospital stay.

CONFABULATION FREE

Is this note free from confabulations/hallucinations?

The note avoids false or fabricated information inconsistent with other chart documentation.

Examples:

© 2025 Small WR et al. *JAMA Network Open*.

- Medications, or procedures, different than those documented elsewhere in the electronic record.
- Vitals, Labs, Radiologic findings, Diagnoses, symptoms and exam findings different than those documented elsewhere in the electronic record.

VI. Few Shot Learning Examples Included in the LLM Prompt

Example Patient 1

Presentation Summary: 61 yo M with DM2, HTN, AF on eliquis presents with SOB and fever for 3 days. He was HDS w/Tmax 101.4, HR 120s, O2 sat 90% on RA which improved to 97% on 2LNC, respiratory rate peaked at 28. On exam, he was diaphoretic in respiratory distress with an irregular HR. Initial WBC was 15.9K. VBG showed mild respiratory alkalosis and negative lactate. Troponin was negative x2. CT angio of the chest showed LUL consolidation and no PE. RVP was negative. EKG showed afib with RVR. He was diagnosed with sepsis from LUL PNA.

Treatment Summary: The patient was initially treated with IV ceftriaxone, PO azithromycin, and 3L LR. Home BP meds were held due to concern for sepsis. His blood cultures returned negative. On hospital day 2, he defervesced, remained hemodynamically stable, converted to sinus rhythm, and was no longer tachycardic or tachypneic. His WBC dropped to 10.2K and need for supplemental oxygen resolved. They experienced hospital acquired complications, sustaining a mechanical fall without head injury as evidenced by negative NCHCT. In addition, EKG showed QTc prolongation to 505, so azithromycin was changed to IV doxycycline.

Resolution Summary: By discharge, the patient no longer required acute care, evidenced by him remaining afebrile, hemodynamically stable, and off oxygen. He was converted to PO doxycycline monotherapy. He was discharged home without extra services and requires 3 additional days of doxycycline along with PCP and cardiology f/u.

Example Patient 2

Presentation Summary: 53 yo F with CAD, CABG presents with chest pain for 6 hours. Patient reports it feels similar to her last heart attack and improved with nitroglycerin. On presentation she was hemodynamically stable, afebrile with stable HR and BP and was well-appearing on exam. She had an abnormal troponin-HS (uptrended from 150 to 500) and EKG with new TWIs but no ST changes. Labs and chest X ray were normal. Transthoracic echocardiogram (TTE) showed normal EF, mild hypokinesis of her lateral LV, and no valve abnormalities. She was admitted for NSTEMI.

Treatment Summary: The patient was initially treated with an aspirin and clopidogrel load and heparin gtt. She was taken for left heart catheterization which found a fully occluded L

© 2025 Small WR et al. *JAMA Network Open*.

circumflex artery, resulting in a stent. Post-procedure her chest pain had resolved, and vital signs were stable. They experienced no hospital acquired conditions or complications.

Resolution Summary: By discharge, the patient no longer required acute care, evidenced by their lack of chest pain and stable vital signs. They were switched back to their home medications which now include daily aspirin and clopidogrel for her new stent. She was discharged home without extra services and plans to follow-up with her primary care provider and cardiology in the next 3 weeks.

Example Patient 3

Presentation Summary: 76 yo F with NICM (EF 30%), severe MR, persistent afib presented with 2 weeks of progressive exertional dyspnea. On presentation, O2 sat was 94% on 6L NC, HR 120, BP 150/95. On exam, she was tachypneic and volume overloaded with 2+ pitting edema in bilateral lower exrtremities and elevated JVP. Chest X-ray revealed pulmonary edema, small bilateral pleural effusions, and no consolidation. WBC wnl, Hb 10.5 (stable from baseline), Cr 1.35 from baseline ~1.1, BNP 1100, troponin-HS 25 -> 23, procalcitonin negative, EKG and transthoracic echocardiogram (TTE) similar to prior. She was admitted for acute decompensated heart failure.

Treatment Summary: The patient was initially treated with furosemide 80mg IV bid and continuation of her home metoprolol and empagliflozin. Her dyspnea resolved, O2 requirement improved to 2L NC, and her AKI resolved by HD3. Her home losartan was switched to sacubitril-valsartan, and spironolactone was started.

Resolution Summary: On hospital day 4, she no longer required supplemental O2 and was walking around the unit comfortably with appropriate afib rate control. Physical therapy deemed her safe for discharge home without increased services. She was discharged at her dry weight 78 kg with instructions for weight-based diuretic dosing. She will have close follow-up with her outpatient PCP and cardiologist including monitoring after switch from ARB to ARNI & new initiation of MRA.

© 2025 Small WR et al. *JAMA Network Open*.

eTable 1. Note Types Included for Summarization, All Remaining Note Types Not Listed Were Considered "Other" Notes

Note Type Name	Categorization
Progress Notes	Main
Consults	Main
Procedures	Main
H&P	Main
H&P (View-Only)	Main
Interval H&P Note	Main
ACP (Advance Care Planning)	Main
Brief Op Notes	Main
Plan of Care	Main
Treatment Plan	Main
Op Notes	Main
Significant Event	Main
Consult Notes	Main
Procedure Notes	Main
Treatment Summary	Main
Pre-Op Note	Main
Post-Op Note	Main
Discharge Narrative	Excluded
OR Nursing	Excluded

© 2025 Small WR et al. *JAMA Network Open*.

Anesthesia Preprocedure Evaluation	Excluded
Anesthesia Postprocedure Evaluation	Excluded
Anesthesia Procedure Notes	Excluded
Hospital Course	Excluded
Telephone Encounter	Excluded
Patient Instructions	Excluded
Committee Review	Excluded
Discharge Instr - AVS First Page	Excluded
Home Health Plan of Care Certification Statement	Excluded
Sedation Documentation	Excluded
Clearance	Excluded
Home Health Certification (Face to Face)	Excluded
Documentation Clarification Note	Excluded
Research	Excluded
HCA Activation Note	Excluded
Dialysis Rounding	Excluded
HEADSS (Age 12-17)	Excluded
Interpreter Information	Excluded
Pt Education - With AVS	Excluded
Pt Education - No AVS	Excluded

© 2025 Small WR et al. *JAMA Network Open*.

eTable 2. Default Parameter Selections Chosen for EHR-Embedded Hospital Course Tool

Parameter	Value
Category: Data Types (Multiple Select)	
Medications	Included
Results	Included
Problem List	Not Included
Other Orders	Not Included
Main Notes	Included
Other Notes	Included
Code Events	Included
Category: Length in Sentences (Select One)	
Unspecified	Included
<10	Not Included
10-20	Not Included
20-30	Not Included
Category: Time Frame for Recent Events (Select One)	
12 Hours	Not Included
24 Hours	Not Included
48 Hours	Not Included
72 Hours	Not Included

© 2025 Small WR et al. *JAMA Network Open*.

Since Admission	Included
Category: Output Format (Select One)	
Narrative	Included
Bullet Points	Not Included
Problem Oriented	Not Included
System-oriented	Not Included
Category – Written for Specialty (Free Text)	No Value
Category - Focus On (Free Text)	No Value
Category – Shorten with Second Pass (Checkbox)	Not Included

© 2025 Small WR et al. *JAMA Network Open*.

eTable 3. Plain Language and Technical Definitions of Editing Metrics

Editing Metric	Plain Language Definition	Technical Definition
Total Edits (Levenshtein Distance)	Number of characters (i.e., letters, numbers, symbols) that differ between the original and edited hospital courses.	Minimum number of character insertions, deletions, substitutions, and translations to convert from one piece of text to another.
Percent Edited (Normalized Levenshtein Distance)	Total edits normalized for the length of the documents being edited.	Total character edits divided by the longer of the pre- and post-edited hospital course in characters.
Semantic Change	Quantify the degree to which the editing process changes the meaning of the original hospital course.	First calculate document embeddings for the original and edited hospital courses. Then calculate cosine similarity (scale 0-1, where 1 means they are identical in meaning). Semantic change is the inverse of cosine similarity, and identifies the degree to which the meaning of the edited hospital course differs from the original hospital course.
Characters Added/Removed	Number of characters that were added to (or removed from) the original hospital course.	Isolate all words added to (or removed from) the original hospital course, then count the characters.

© 2025 Small WR et al. *JAMA Network Open*.

eTable 4. Guide to Interpreting Editing Statistics: Scenarios and Corresponding Statistical Patterns

Revision Scenario	Example Sentence (Original → Revised)	Cognitive Effort of Editing	Characters Edited (% Edited)	Semantic Change in Editing
Minor Revision	"Patient was started on antibiotics." → "The patient was started on antibiotics."	Low, minimal cognitive load	Low (less than 10% edited)	Low (less than 10% difference from original)
Targeted Meaning-Altering Edits	"The patient tolerated the procedure well." → "The patient did not tolerate the procedure well."	Moderate, requires contextual awareness	Low (less than 10% edited)	Moderate-High (more than 10% difference from original)
Paraphrase	"IV fluids were given to the patient due to dehydration." → "Due to dehydration, IV fluids were administered to the patient."	Moderate, involves syntactic restructuring	Moderate-High (more than 10% edited)	Low (less than 10% difference from original)
Major Revision	"The patient was monitored overnight." → "The patient was admitted to the ICU for continuous monitoring after 3 units packed red blood cells."	High, demands significant cognitive resources for thorough content revision	Moderate-High (more than 10% edited)	Moderate-High (more than 10% difference from origina)

© 2025 Small WR et al. *JAMA Network Open*.

eTable 5. Percentage Rater Agreement on the 4Cs HC Quality Standard Survey Questions

	Exact Agreement	Agreement within 1 Likert Point
Complete	26%	78%
Concise	27%	76%
Cohesive	26%	63%
Confabulation-Free	47%	88%

© 2025 Small WR et al. *JAMA Network Open*.

eFigure 2. Reviewer Score Combinations on Each of the 4Cs HC Quality Standard Survey Questions – pure agreement denoted by bolded cells of the contingency table.

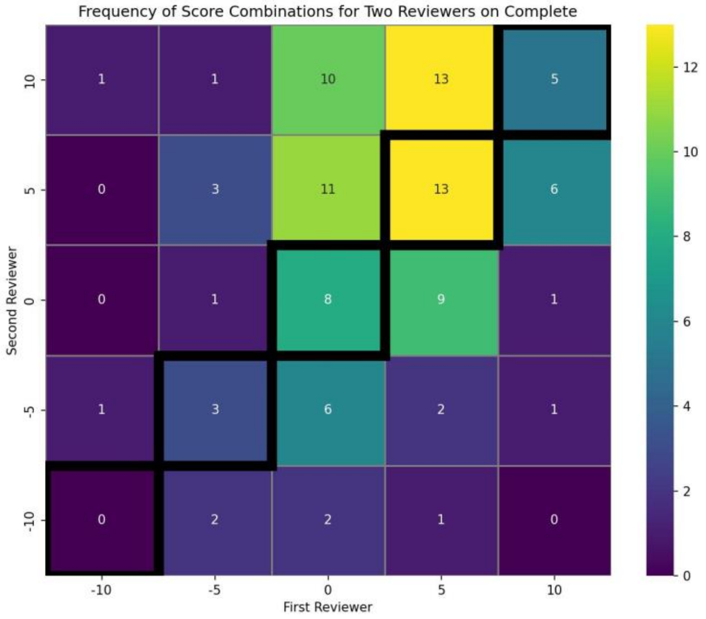

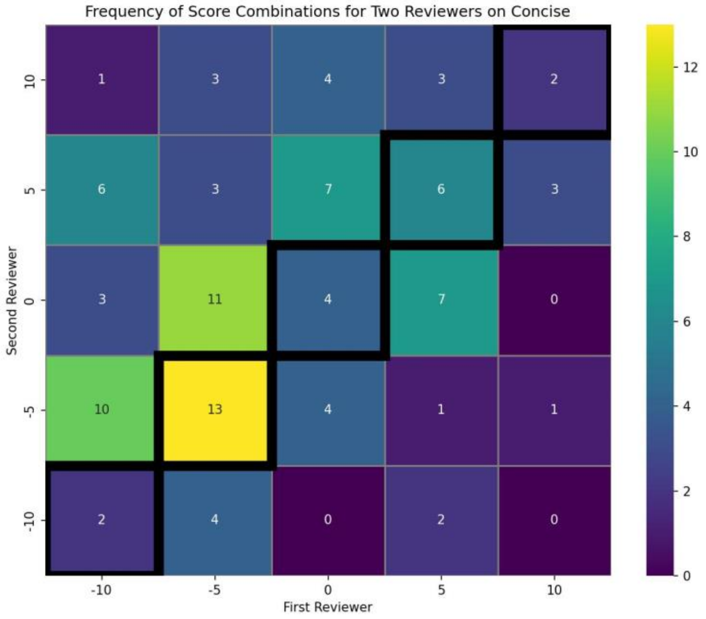

© 2025 Small WR et al. *JAMA Network Open*.

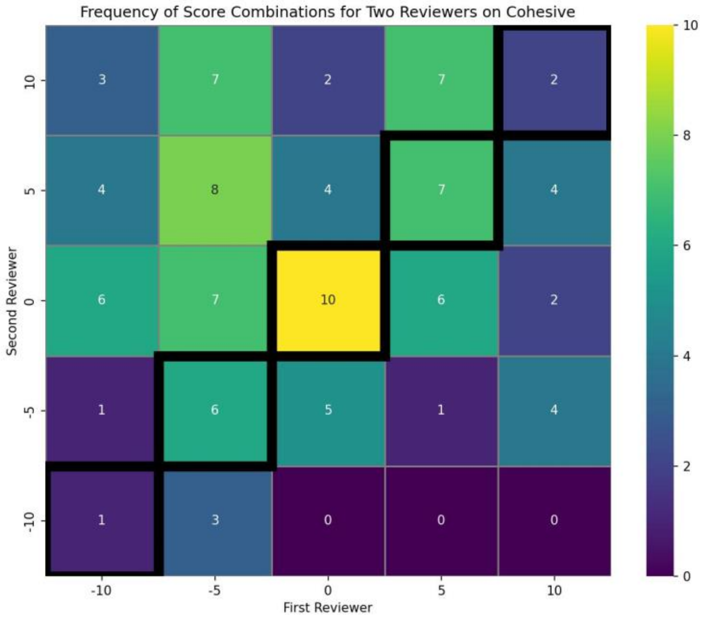

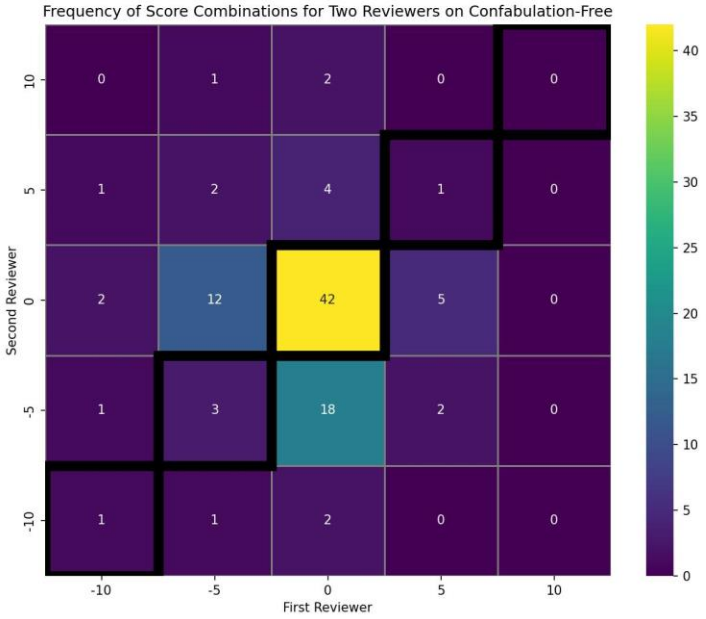

© 2025 Small WR et al. *JAMA Network Open*.
