# Supplementary material for: Evaluating Hospital Course Summarization by an Electronic Health Record–Based Large Language Model
Source: JAMA Netw Open. 2025 Aug 13;8(8):e2526339. doi: 10.1001/jamanetworkopen.2025.26339 (PMC12351420; doi:10.1001/jamanetworkopen.2025.26339)
Supplement: Supplement 2. — Data Sharing Statement [file jamanetwopen-e2526339-s002.pdf]

# Data Sharing Statement

Small. Evaluating Hospital Course Summarization by an Electronic Health Record-Based Large Language Model. *JAMA Netw Open*. Published August 13, 2025. doi:10.1001/jamanetworkopen.2025.26339

**Data**
**Data available:** No
